# Sound Power Estimation for Beam and Plate Structures Using Polyvinylidene Fluoride Films as Sensors

**DOI:** 10.3390/s17051111

**Published:** 2017-05-16

**Authors:** Qibo Mao, Haibing Zhong

**Affiliations:** 1 School of Mechanical Engineering, Yangzhou University, Yangzhou 225127, China; 2 School of Aircraft Engineering, Nanchang Hang Kong University, Nanchang 330063, China; 3 Kuang-Chi Advanced Institute of Technology, Shenzhen 518000, China; haibin.zhong@kuang-chi.com or zhb.0429@foxmail.com

**Keywords:** PVDF-based radiation mode, sound power, PVDF film, beam, plate

## Abstract

The theory for calculation and/or measurement of sound power based on the classical velocity-based radiation mode (V-mode) approach is well established for planar structures. However, the current V-mode theory is limited in scope in that it can only be applied to conventional motion sensors (i.e., accelerometers). In this study, in order to estimate the sound power of vibrating beam and plate structure by using polyvinylidene fluoride (PVDF) films as sensors, a PVDF-based radiation mode (C-mode) approach concept is introduced to determine the sound power radiation from the output signals of PVDF films of the vibrating structure. The proposed method is a hybrid of vibration measurement and numerical calculation of C-modes. The proposed C-mode approach has the following advantages: (1) compared to conventional motion sensors, the PVDF films are lightweight, flexible, and low-cost; (2) there is no need for special measuring environments, since the proposed method does not require the measurement of sound fields; (3) In low frequency range (typically with dimensionless frequency *kl* < 4), the radiation efficiencies of the C-modes fall off very rapidly with increasing mode order, furthermore, the shapes of the C-modes remain almost unchanged, which means that the computation load can be significantly reduced due to the fact only the first few dominant C-modes are involved in the low frequency range. Numerical simulations and experimental investigations were carried out to verify the accuracy and efficiency of the proposed method.

## 1. Introduction

Calculation or measurement of sound power is an important issue in structure-borne sound problems, since sound power is a global quantity to characterize the strength of the sound generated from a vibrating structure. Measurement techniques for estimating the sound power of vibrating structures play important roles for the researcher who wishes to understand their acoustic behaviour and to control their sound radiation. However, there are some problems to be solved: the standard methods are based on pressure measurement or based on sound intensity measurement [1,2]. These methods always require special acoustic environments (such as anechoic rooms or reverberation rooms). 

To solve these problems, the use of structural motion sensors (such as accelerometers and laser vibrometers) to estimate sound power has attracted more and more attention in recent years. It has been widely accepted that the radiation mode approach is a powerful tool for interpreting sound radiation [3,4,5,6,7]. The original idea of the radiation mode approach can be found in a seminal study by Borgiotti [3], who obtained a set of orthonormal boundary velocity patterns through singular value decomposition (SVD). These patterns can be divided into efficient and inefficient velocity distributions. Elliott and Johnson [4] and Cunefare and Currey [5] defined these velocity patterns as “radiation mode”. The radiation modes are defined as a set of velocity distributions. Each radiation mode represents a possible radiation pattern and has independent radiation efficiency. At low frequencies, the first radiation mode accounts for the majority of the sound power. Pasqual et al. [6] applied radiation modes to the problem of sound field synthesis by a spherical loudspeaker array. An important result of their work is that the array with preprogrammed surface velocity distributions corresponding to its acoustic radiation modes is a better control strategy than using spherical harmonics as elementary directivities. Chanpheng et al. [7] applied radiation modes for a highway bridge structural acoustics control problem. They concluded that the significant radiation mode shapes at maximum frequency of interest can be used in controller design and as guidelines for positioning actuators and sensors. Tao and Qiu [8] compared the velocity sensors required to estimate sound power by using radiation modes or vibration modes for the same level of accuracy for low damped baffled simply supported rectangular plates. They concluded that employing radiation mode requires fewer sensors below the first resonance frequency, while employing vibration modes at structural resonance frequencies requires fewer sensors than that employing radiation modes. Bai and Tsao [9] measured sound power using laser displacement sensor and classical radiation mode approach for a baffled point-driven flexible plate. In comparison to the conventional pressure-based ISO 3745 method [1], it is shown that this technique produced more accurate estimations of sound power. Ji and Bolton [10,11] extended the classical radiation modes to structure-dependent radiation modes which are defined as a set of modal velocity distributions. Yamaguchi et al. [12] proposed the force radiation mode concept to understand the relationship between sound power and driving force distribution. Lu et al. [13] proposed a mapped radiation mode theory to avoid the cumbersome computation of radiation mode for convex structures. They developed an efficient method to compute sound power by keeping advantages of the radiation mode and avoiding the numerical difficulties in the computation of radiation modes, especially for wideband frequency and large scale problems.

So far, sound power estimation based on radiation mode approach was limited to using the surface velocity distribution information, so the present classical or extended radiation mode theories are termed as velocity-based radiation modes (V-modes) in this study. 

Piezoelectric materials have attracted more and more attention as structural sensors in structural vibration and acoustic problems [14,15,16,17,18,19,20,21,22,23]. For examples, Chen and Wang [14] used a rectangularly shaped polyvinylidene fluoride (PVDF) film as a sensor to perform structural modal testing. They experimentally verified that the modal parameters (such as natural frequencies, damping ratios and mode shapes) can be properly identified for the cantilever beam and simply supported plate. Oliveira et al. [15] also estimated the modal parameters of composite flat plate models through experimental modal analysis using PVDF films as sensors. The PVDF experimental results were verified by the results from laser Doppler vibrometry. The cross-sensitivity and size effects of PVDF film sensors on modal testing were experimental investigated by Chuang et al. [16]. Luo et al. [17] presented a real-time nonintrusive method for deflection monitoring by using PVDF films.

PVDF sensors can also be designed by shaping the surface electrode [18], whereby the output of the sensor can be made sensitive to selected modal coordinates, and other modal coordinates may be filtered out. PVDF sensors are continuous sensors and thus avoid spatial aliasing problems. The design of modal sensors using shaped PVDF film can be traced back to Lee and Moon [18]. One of the most recent motivations has been the application of active vibration control (AVC) and active structural acoustic control (ASAC). Different design approaches, such as parametric level set method [19], differential transformation method [20], were imposed to the shaped PVDF modal sensors for different structures. Several examples for the design of AVC system and ASAC system by using PVDF sensors are described in [21,22,23,24]. Reviews of the recent work on piezoelectric sensors for active control systems are available in [23,24] and are not duplicated here. 

It is well known that fewer modes are required by employing the radiation mode approach in low-frequency sound power calculation. However, no literature has ever discussed how to measure the sound power by using rectangular piezoelectric films. Therefore, a method to estimate sound power based on piezoelectric sensors has yet to be developed.

This study presents a technique to estimate sound power by using a piezoelectric film. In this study, segments of piezoelectric film made of PVDF are chosen because it adds little loading on light structures, and is easy to cut into the desired segments [17,18]. Other advantages of PVDF film sensors are high flexibility, low cost, and high mechanical strength to endure physical impacts. In order to estimate the sound power using PVDF film, a new concept of PVDF-based radiation mode approach is introduced to describe the relationship between sound power and the output signals of PVDF films of the a vibrating structure. Due to the current output of PVDF used, the PVDF-based radiation mode is termed as C-mode. The proposed method is a hybrid of vibration measurement and numerical calculation of C-modes, because sound power of the vibrating structures is estimated by proposed C-mode approach combined with measurement of PVDF film signals. Finally, with examples of a clamped-clamped beam and a clamped plate, the proposed sound power measurement techniques are verified by numerical simulation as well as experiments.

## 2. Sensing Principle of PVDF Film 

**Figure 1:**
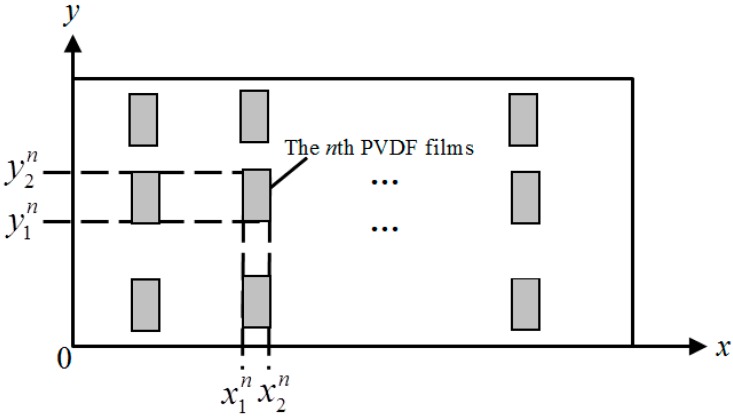
Rectangular PVDF films bonded on a plate.

The sensing principle of PVDF film has been clearly introduced by Lee and Moon’s work [18]. It is briefly reviewed here for completeness. Consider a plate with length *L_x_*, width *L_y_* and thickness *h*. *N* rectangular PVDF film patches (same size) are equally attached on the top surface, as shown in Figure 1.

Assume that the direction of maximum stress/charge coefficient *e*_31_ is parallel with *y*-axis, the output current of the *n*th PVDF sensor can be expressed as follows [18]:

(1) $$I_{n}=\frac{h+h_{f}}{2}\int_{\;x_{1}^{n}}^{\;x_{2}^{n}}\int_{y_{1}^{n}}^{y_{2}^{n}}\left(e_{32}\frac{\partial^{2}v\left(x,y\right)}{\partial x^{2}}+e_{31}\frac{\partial^{2}v\left(x,y\right)}{\partial y^{2}}\right)dydx$$

where *h_f_* is the PVDF sensor thickness. *e*_31_ and *e*_32_ are the PVDF sensor stress/charge coefficients. $x_{1}^{n}$, $x_{2}^{n}$, $y_{1}^{n}$ and $y_{2}^{n}$ are the edge coordinates of the *n*th PVDF sensor. *v* (*x*, *y*) is the velocity of the plate.

The velocity distribution of the plate can be represented by a series of expansion:

(2) $$v\left(x,y\right)=\sum_{m=1}^{M}A_{m}\Phi_{m}\left(x,y\right)=\Phi^{T}A$$

where *Φ_m_*(*x*, *y*) and *A_m_* represent the *m*th structural mode shape and modal velocity, respectively. *m = (m_x_*, *m_y_)* denote the index of structural mode in the *x*- and *y*-axis, respectively. ***Φ*** and ***A*** are *M* × 1 vectors. Superscript T is denoted transpose.

Substituting Equation (2) into Equation (1), the output current of the *n*th PVDF sensor is given by:

(3) $$I_{n}=\frac{h+h_{f}}{2}\int_{x_{1}^{n}}^{x_{2}^{n}}\int_{y_{1}^{n}}^{y_{2}^{n}}\left(e_{31}\sum_{m=1}^{M}A_{m}\frac{\partial^{2}\Phi_{m}\left(x,y\right)}{\partial x^{2}}+e_{32}\sum_{m=1}^{M}A_{m}\frac{\partial^{2}\Phi_{m}\left(x,y\right)}{\partial y^{2}}\right)dydx$$

Equation (3) can be rewritten into matrix form:

(4) $$I=\sum_{m=1}^{M}F_{m,n}A_{m}=KA$$

with $K_{m,n}=\frac{h+h_{f}}{2}\int_{x_{1}^{n}}^{x_{2}^{n}}\int_{y_{1}^{n}}^{y_{2}^{n}}\left(e_{31}\sum_{m=1}^{M}\frac{\partial^{2}\Phi_{m}\left(x,y\right)}{\partial x^{2}}+e_{32}\sum_{m=1}^{M}\frac{\partial^{2}\Phi_{m}\left(x,y\right)}{\partial y^{2}}\right)dydx$.

## 3. Radiation Modes Theory 

### 3.1. V-Mode Approach

Consider a baffled, finite rectangular plate. The plate is divided into *N* elements with equal area. The vector of sound pressure immediately in front of each of these elements is denoted as ***p***, and the vector of normal velocities of these elements is denoted as ***v***, then we have:

(5) p=Zv

where ***Z*** is the matrix of impedance relating the sound pressure to the normal velocity for each element [4]. For radiators in a baffle the matrix ***Z*** can be written as [4,9]:

(6) Z=ρoco[1−exp(ikΔSπ)−ikΔS2πexp(ikr12)r12...−ikΔS2πexp(ikr1N)r1N−ikΔS2πexp(ikr21)r211−exp(ikΔSπ)...−ikΔS2πexp(ikr2N)r2N............−ikΔS2πexp(ikrN1)rN1−ikΔS2πexp(ikrN2)rN2...1−exp(ikΔSπ)]

where *ρ_o_* and *c_o_* are the density and the sound speed of the acoustic medium (air in this study), respectively. Δ*S* denotes the elemental area. *k* = *ω*/*c_o_* is the wave number, and *ω* is the angular frequency of the plate. *r_mn_* = *r_nm_* is the distance from element *m* to element *n* $i=\sqrt{-1}$.

As discussed in Refs [3,4,5,6], the sound radiation can be assumed to be due to a number of elemental radiators. The sound power can be expressed as [4]:

(7) W=(ΔS2)Re[vHp]

where the superscript H denotes the complex conjugate transpose. Re denotes the real part of the bracketed quantity.

Substituting Equation (5) into Equation (7), the radiated sound power can be formulated as:

(8) W=ΔS2Re[vHZv]=vHRv

where R=ΔS2Re(Z), and ***R*** is the radiation mode matrix, which is real, symmetrical, positive definite, and the (*m*, *n*) element of matrix ***R*** is $R_{mn}=\frac{\omega^{2}\rho_{o}{\left(\Delta S\right)}^{2}}{4\pi c_{o}}\frac{\sin\left(kr_{mn}\right)}{kr_{mn}}$. 

From Equation (8), it can be found that, if the velocity distribution of the plate is measured, the sound power can be obtained, so in this study we call this classical radiation mode the velocity-based radiation mode (V-mode).

### 3.2. C-mode approach

Notice that the main goal of this study is to estimate the sound power using PVDF film. Assume that the total number of structural mode is equal to the number of PVDF sensor, ***K*** matrix in Equation (4) becomes square, and the modal velocity can be expressed by PVDF current output vector ***I***:

(9) $$A=K^{-1}I$$

According to Equation (2), the velocity distribution vector ***v*** in Equation (8) can be rewritten as:

(10) $$v={\tilde{\Phi }}^{T}A$$

where $\tilde{\Phi }$ is a *M* × *N* structural mode shape matrix with the *m*th column ${\tilde{\Phi }}_{m}$ representing the amplitudes of the *m*th mode shape for *N* elements of the plate.

Substituting Equation (9) into Equation (10), the velocity distribution can be expressed as a function of the PVDF sensor outputs:

(11) $$v={\tilde{\Phi }}^{T}K^{-1}I$$

Substituting Equation (11) into Equation (8), the sound power can be rewritten as:

(12) $$W={\left({\tilde{\Phi }}^{T}K^{-1}I\right)}^{H}R\left({\tilde{\Phi }}^{T}K^{-1}I\right)=I^{H}{\left({\tilde{\Phi }}^{T}K^{-1}\right)}^{H}R\left({\tilde{\Phi }}^{T}K^{-1}\right)I=I^{H}MI$$

where $M={\left({\tilde{\Phi }}^{T}K^{-1}\right)}^{H}R\left({\tilde{\Phi }}^{T}K^{-1}\right)$, is a real positive definite matrix.

Similar to classical V-mode theory, the matrix ***M*** can be diagonalized through orthogonal transformation and written as:

(13) $$M=Q\Lambda Q^{T}$$

where ***Λ*** is an *N* × *N* diagonal matrix, whose elements, λ*_i_*, are eigenvalues of ***M***.

Clearly, the eigenvalues $\lambda_{i}$ in Equation (13) is real and positive with $\lambda_{1}\geq \lambda_{2}\geq \lambda_{3}\geq \ldots \ldots \geq \lambda_{N}>0$. The corresponding eigenvector ***Q****_i_* are orthogonal one another. Because eigenvectors matrix ***Q*** is real, $Q^{T}=Q^{H}$, we can rewrite sound power *W* as:

(14) $$W={\left(Q^{T}I\right)}^{H}\Lambda \left(Q^{T}I\right)=y^{H}\Lambda y=\sum_{i=1}^{N}\lambda_{i}{\left|y_{i}\right|}^{2}$$

where $y=Q^{T}I$.

From Equation (14), it can be found that the sound power can be described as a summation of a set of uncoupled radiation terms. Notice that the sound power estimated by Equation (14) is based on PVDF film output signals. For this reason, the eigenvector ***Q****_i_* is termed PVDF-based radiation mode (C-mode). Similar to V-mode approach [8], C-mode approach is able to give accurate estimation of sound power only in the frequency range involved the first *N* structural modes if *N* number of PVDF films are used.

## 4. Numerical Calculations

In this section, the sound power using C-mode approach is estimated numerically. The theory mentioned in the previous section is applied to two baffled finite structures (one clamped-clamped beam and one clamped rectangular plate) in free space. In particular, the contributions of the C-modes to the sound power; the shapes of the C-modes; the approximation error of the C-mode order reduction, are simulated.

In all of the cases, the dynamics of the PVDF sensors are not modeled, because the PVDF sensor thickness *h_p_* is much smaller than structures thickness *h*, the mass and stiffness of the sensor is then negligible compared to the properties of the structures. This is a reasonable assumption since the PVDF sensor thickness is typically 28 µm to 110 µm.

The dimensions of the beam and plate for calculation are 500 mm × 30 mm × 3.5 mm and 440 mm × 340 mm × 2 mm, respectively. Young’s modulus, Poisson ratio, loss factor and density of both structures are 70 × 10^9^ Pa, 0.3, 0.01 and 2700 kg/m^3^, respectively. The physical properties of the PVDF film used in calculation are listed in Table 1.

### 4.1. Sound Power for Beam Structure

Firstly, the sound power from a baffled clamped-clamped beam in free space is studied. Because $L_{x}>>L_{y}>>h$, so response change along the width of the beam can be neglected. And Equation (1) can be simplified as:

(15) $$I_{n}=\frac{h+h_{f}}{2}\left(y_{2}^{n}-y_{1}^{n}\right)\int_{\;x_{1}^{n}}^{\;x_{2}^{n}}\left(e_{31}\frac{\partial^{2}v\left(x,y\right)}{\partial x^{2}}\right)dx$$

The mode shape for clamped-clamped beam can be expressed as:

(16) $$\Phi_{n}\left(x\right)=\cosh\left(\lambda_{n}x\right)-\cos\left(\lambda_{n}x\right)-\beta_{n}\left[\sinh\left(\lambda_{n}x\right)-\sin\left(\lambda_{n}x\right)\right]$$

where $\beta_{n}=\frac{\cosh\left(\lambda_{n}L_{x}\right)-\cos\left(\lambda_{n}L_{x}\right)}{\sinh\left(\lambda_{n}L_{x}\right)-\sin\left(\lambda_{n}L_{x}\right)}$ and *λ_n_* is the root for the equation cosh(λ) cos(λ) = 1.

**Figure 2:**
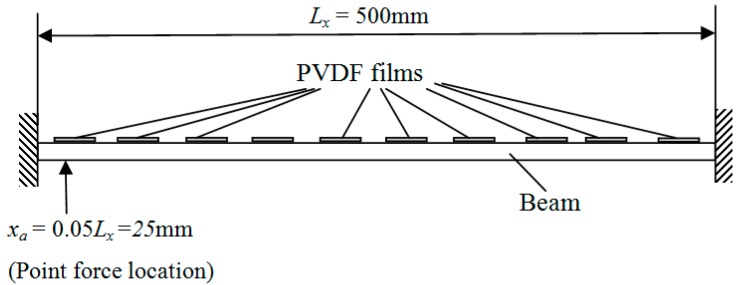
The locations of PVDF films and excitation point force on the beam.

Assume that 10 rectangular PVDF film segments are equally attached on the surface. The total number of C-modes is then 10, as is clear from Equations (14) and (15). Moreover, the dimensionless frequency *kl* is defined as the acoustic wave number *k* multiplied by the length of the beam *L_x_* [4,8]. The PVDF output currents are assumed to be ideally measured, a point excitation force is applied at an arbitrarily chosen location ( *x_a_* = 0.05*L_x_*), the locations of PVDF films and excitation point force are shown in Figure 2.

**Figure 3:**
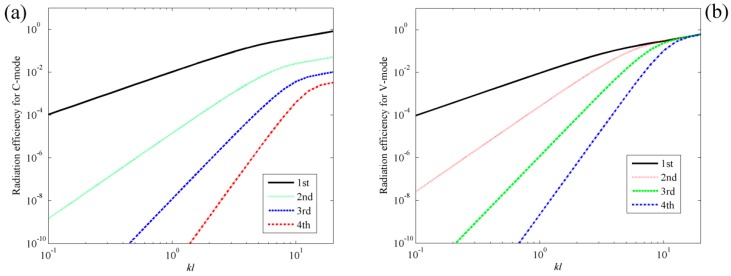
The radiation efficiencies of beam for (**a**) the first four C-modes; (**b**) the first four V-modes.

Figure 3a shows the radiation efficiencies for the first four C-modes and the first four V-modes. For comparison, the radiation efficiencies for V-mode are also presented in Figure 3b. A detailed description of the radiation efficiencies for V-mode can be found in [4,5,6,7,8]. From Figure 3a, it can be found that at lower dimensionless frequency ranges (0 < *kl* < 4), the radiation efficiency of the first C-mode is significant and the radiation efficiencies of the other C-modes are almost negligible. At higher dimensionless frequency ranges (4 < *kl* < 20), the radiation efficiency of the first mode is still significant, but the radiation efficiencies of the other modes, especially the second and third C-modes, increase in relation to the dimensionless frequencies. This means that the sound power can be estimated by using the first few C-modes in low frequency range. Comparing Figure 3a to Figure 3b, it can be found that the radiation efficiency for C-mode decreases more sharply than V-mode as the mode order increases, which indicates that less C-modes are required for the same level of accuracy in the prediction of sound power, if the mode amplitudes of all the modes are equal.

**Figure 4:**
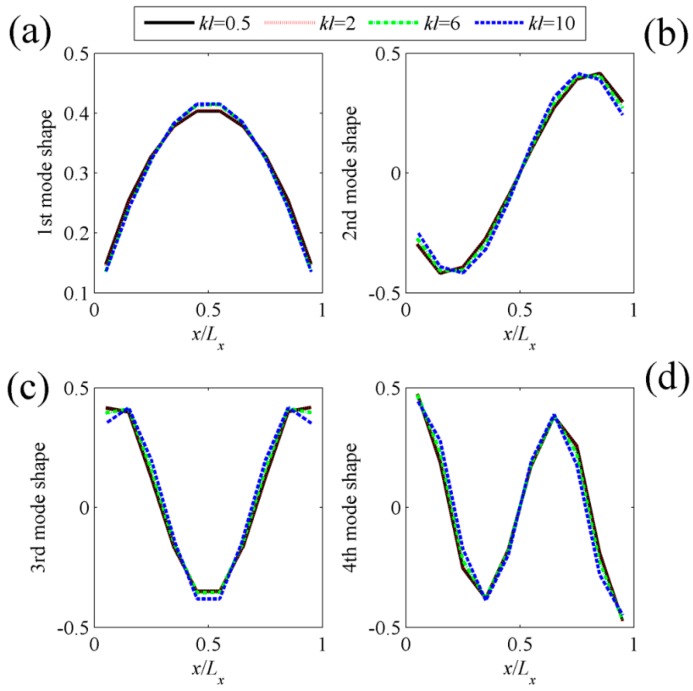
The shapes of (**a**) the first; (**b**) the second; (**c**) the third; (**d**) the fourth C-modes for clamped-clamped beam at different dimensionless frequencies.

**Figure 5:**
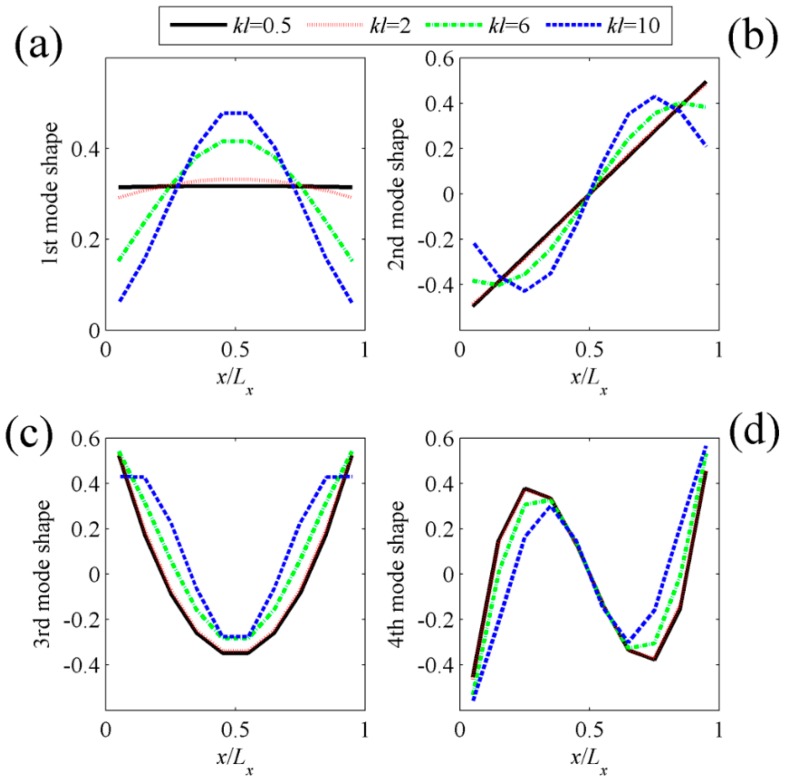
The shapes of (**a**) the first; (**b**) the second; (**c**) the third; (**d**) the fourth V-modes for clamped-clamped beam at different dimensionless frequencies.

Figure 4 shows the shapes of the first four C-modes, that is, the eigenvectors derived from Equation (13), under different dimensionless frequencies. In principle, the C-mode shapes are frequency-dependent, as shown in Equation (13). However, in the quite large dimensionless frequency ranges (*kl* < 10), the C-mode shapes (eigenvectors) vary slowly with respect to frequency. It means that we can select a frequency independent C-mode **Q***_f_* at a low frequency to represent the frequency dependent C-mode ***Q*** in this dimensionless frequency range. So the computation load can be reduced significantly. However, the V-mode shapes, as shown in Figure 5, are more sensitive to frequency. The detailed calculation method for V-mode shapes can be found in [4,6].

From the discussion above, it can be found that, compared with the V-mode approach, the C-mode approach has two advantages. First, the radiation efficiency decreases much quickly as the mode order increases. Second, the shapes of C-modes are almost frequency independent. 

According to above analysis, the sound power can be estimated by using first *L* frequency independent C-modes **Q***_f_*, yields:

(17) $$W\approx \sum_{i=1}^{L}\lambda_{i}{\left|{\hat{y}}_{i}\right|}^{2}$$

where ${\hat{y}}_{i}=Q_{f}^{T}I$ is the *i*th C-mode amplitude, and *L* < *N*.

**Figure 6:**
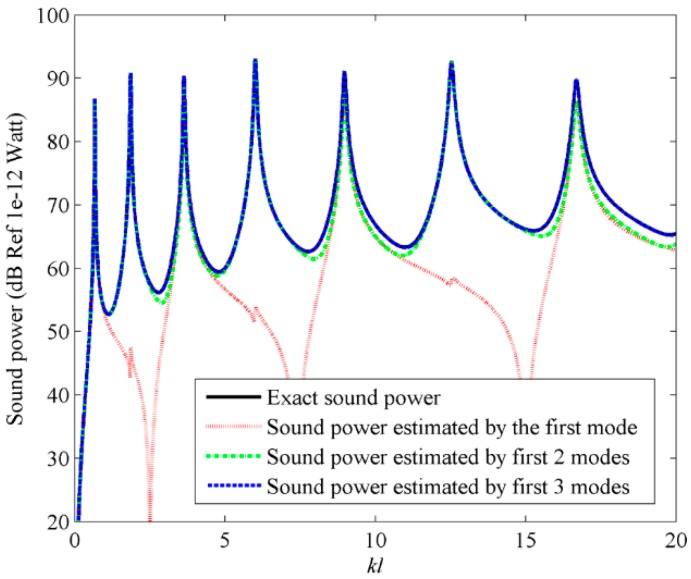
The sound power for beam calculated by using the frequency dependent C-modes.

Figure 6 shows the sound power obtained by using the frequency dependent C-modes. The exact sound power is calculated by using Equation (8). From Figure 6, it can be found that sound power can be accurately obtained if the first three frequency dependent C-modes are used.

**Figure 7:**
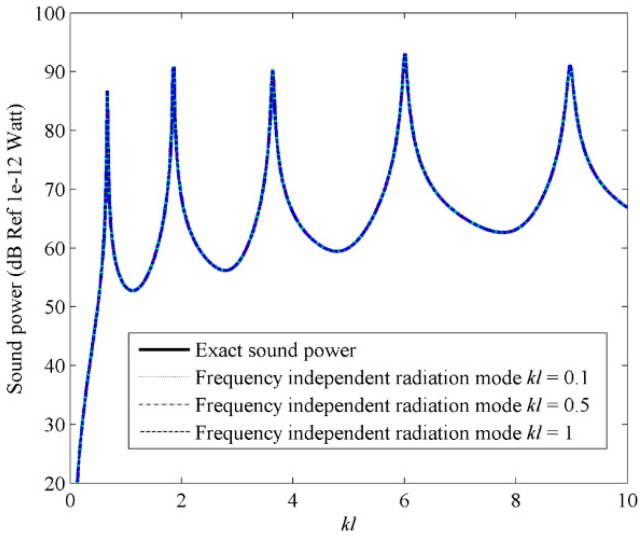
The sound power for beam calculated by using the first three frequency independent C-modes.

The computation load can be significantly reduced if the frequency independent C-modes in Equation (17) are used to estimate the sound power. One question is how to select the frequency independent C-mode **Q***_f_* to represent the frequency dependent C-mode ***Q***. To answer this question, the sound power using the first three frequency independent C-modes is calculated, as shown in Figure 7. 

**Figure 8:**
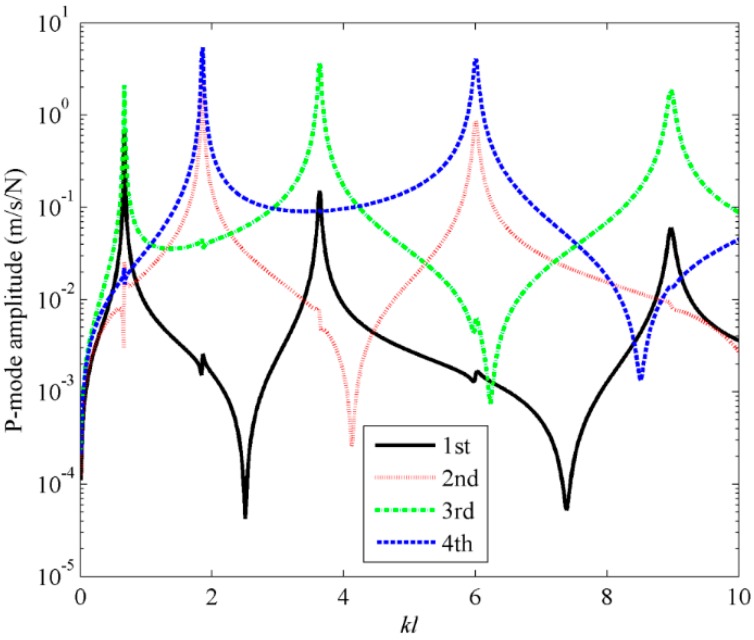
The first four C-mode amplitudes for beam under the point force excitation.

**Figure 9:**
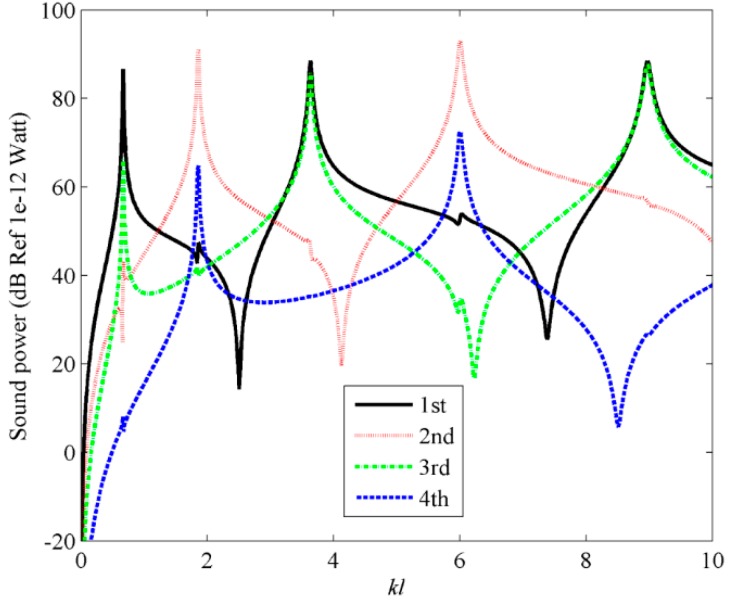
The sound power of the first four C-modes for beam under the point force excitation.

Mode shapes at four fixed dimensionless frequencies with *kl* = 0.1, 0.5, 1 and 2 are selected to estimate the sound power. From Figure 7, it is found that the results using different frequency independent C-modes coincides with each other, so only a single dashed curve can be found in the figure. It means that the mode shape at any low dimensionless frequency can be used as the frequency independent radiation mode **Q***_f_*. Hence, it is suggested in this specific model that, within the range of the dimensionless frequency, 0 < *kl* < 10, the sound power can be estimated by using the first three frequency independent C-modes. From Equation (17), it can be found that both the radiation efficiency and the C-mode amplitude need to be considered in the sound power calculation. Figure 8 and Figure 9 show the first four C-mode amplitudes and the corresponding modal sound power. From these figures it can be found that the odd/even C-modes only contain the contribution of the odd/even structural modes. Furthermore, the sound power due to the 4th C-mode is negligible compared to the other C-modes, though the amplitude of the 4th C-mode is quite large. This is because the radiation efficiency of the 4th C-mode is very low (as shown in Figure 3a).

**Figure 10:**
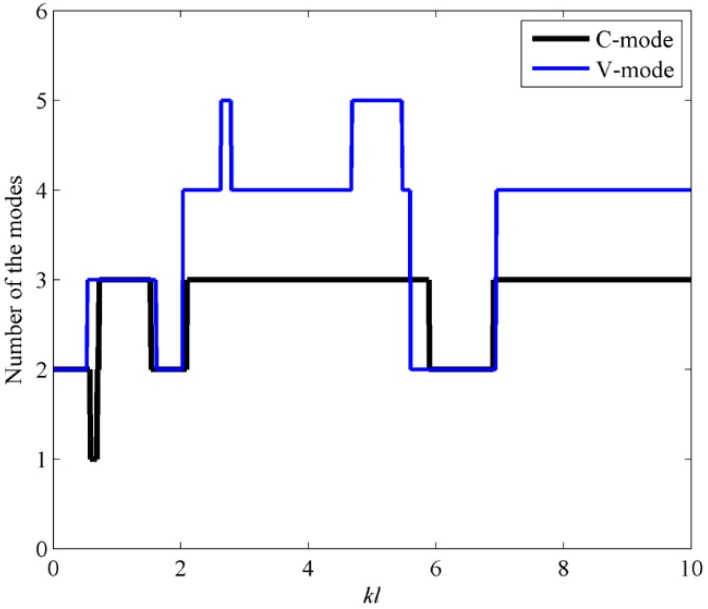
The mode number requirements by C-mode and V-mode to account for 99% of the sound power when the beam is excited by the point force.

Assuming the prediction precision of the sound power must exceed 99%, the mode number requirements of using C-mode and V-mode are calculated and the results are presented in Figure 10. From Figure 10, it can be found that, compared to V-modes, less C-modes are required for the same level of accuracy in the prediction of sound power.

### 4.2. Sound Power for Plate Structure

To further investigate the acoustic properties of the proposed C-mode approach, a baffled clamped plate is chosen for calculation. The mode shape functions for clamped plate can be expressed as the product of two independent beam functions:

(18) $$\Phi_{mn}\left(x,y\right)=X_{m}\left(x\right)\cdot Y_{n}\left(y\right)$$

where *X_m_*(*x*) and *Y_n_*(*y*) can be chosen by using Equation (16), such as *X_m_*(*x*) = *Φ_m_*(*x*) and *Y_n_*(*y*) = *Φ_n_*(*y*).

**Figure 11:**
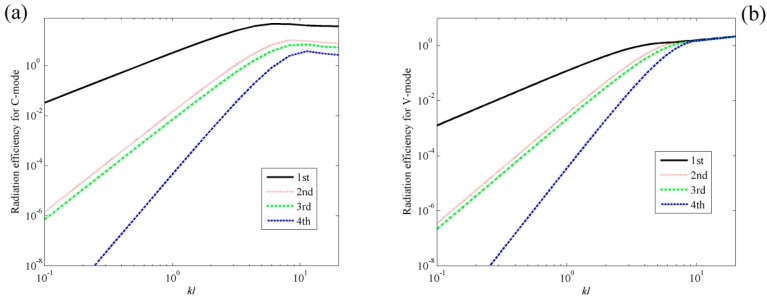
The radiation efficiencies of plate for (**a**) the first four C-modes; (**b**) the first four V-modes.

**Figure 12:**
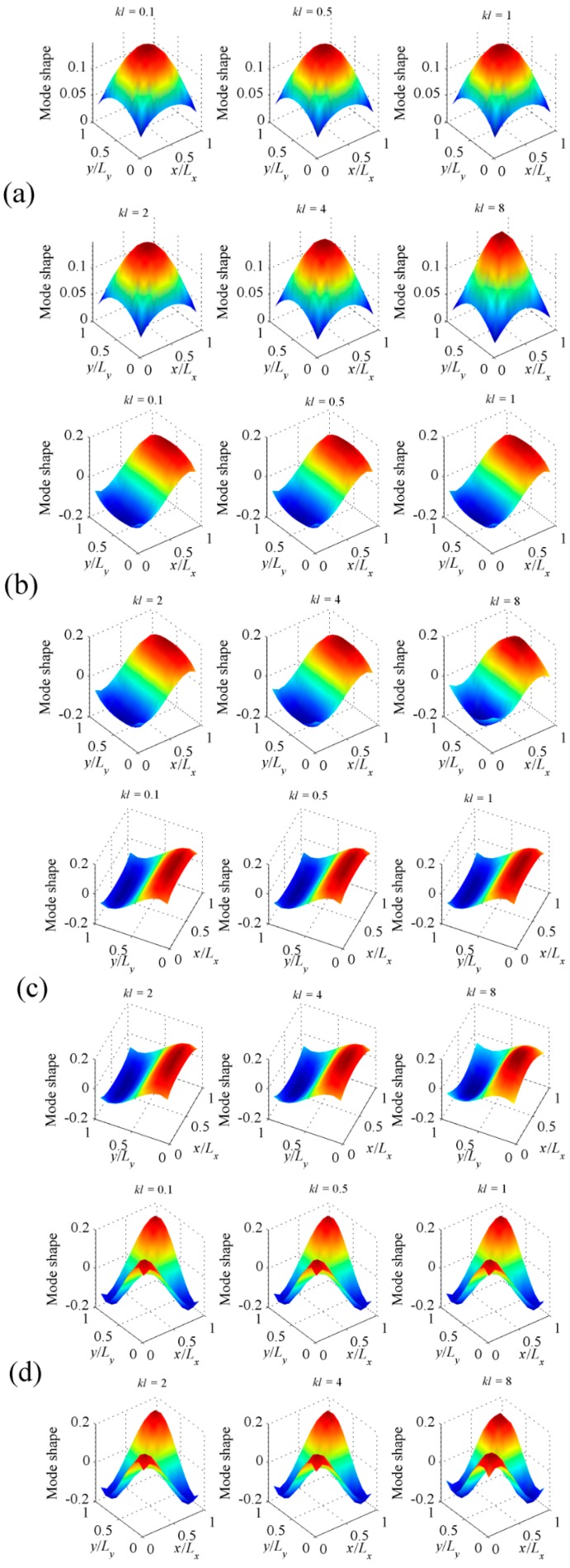
The shapes of (**a**) the first; (**b**) the second; (**c**) the third; (**d**) the fourth C-modes for clamped plate at different dimensionless frequencies.

Assume the 10 × 10 rectangular PVDF sensor equally attached on the surface of the plate. The total number of C-modes is then 100, as is clear from Equations (13) and (14). Figure 11a shows the radiation efficiencies of the first four C-modes, that is, the largest four eigenvalues derived from Equation (13). The radiation efficiencies of V-modes are also presented in Figure 11b for comparison purposes. Similar to beam case, it can be found that the radiation efficiency for C-mode decreases more sharply than V-mode as the mode order increases. Figure 12 shows the shapes of the first four C-modes, that is, the eigenvectors derived from Equation (13), under different dimensionless frequencies. Similar to beam case, the C-mode shapes (eigenvectors) remain almost unchanged with respect to frequency. It means that the C-modes shapes in quite large frequency range (*kl* < 8 in this case) can be seen as frequency independent. It means that we can select any *kl* < 8 frequency independent C-mode to represent the frequency dependent C-mode in this dimensionless frequency range. Equation (17) can also be used to estimate the sound power for the plate case.

**Figure 13:**
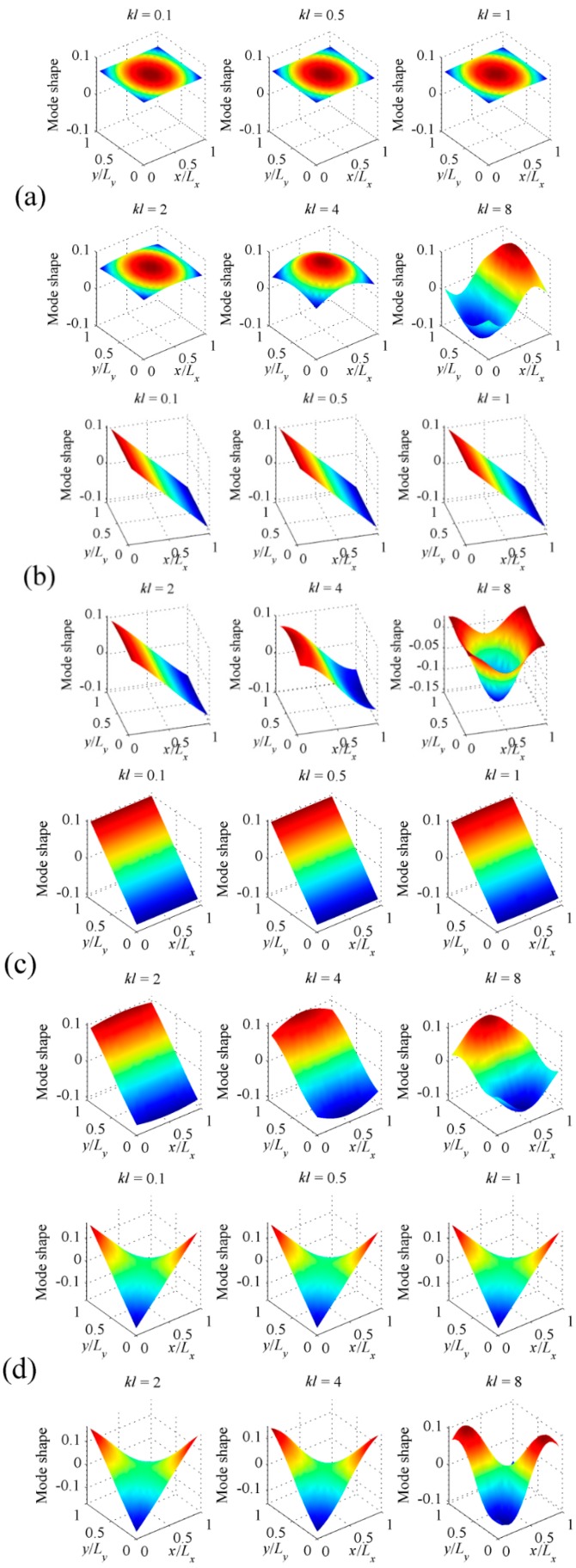
The shapes of (**a**) the first; (**b**) the second; (**c**) the third; (**d**) the fourth V-modes for clamped plate at different dimensionless frequencies.

To further investigate the difference between C-mode and V-mode, the first four V-mode shapes under different frequencies are also presented in Figure 13. At low frequencies (*kl* < 1), the V-mode shapes can be considered as frequency independent, as expected. However, as frequency increase, the V-mode shapes are more and more sensitive to frequency, as shown in Figure 13. 

**Figure 14:**
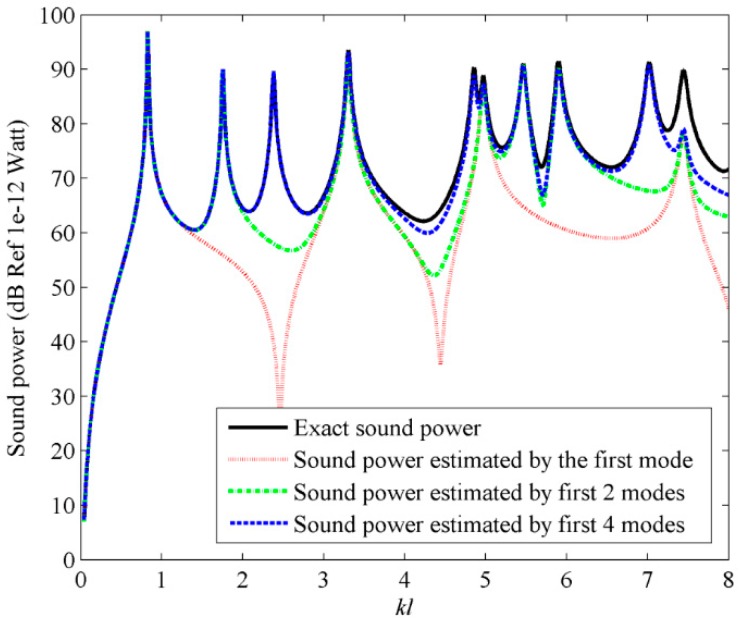
The sound power for plate calculated by using the first four frequency independent C-modes.

To calculate the sound power, a point excitation force is applied at an arbitrarily chosen location (*x_a_* = 0.06*L_x_*, *y_a_* = 0.06*L_y_*). Figure 14 shows the sound power obtained by using the frequency independent C-modes, with a fixed dimensionless frequency with *kl* = 0.1. From Figure 14, it can be found that using the first four C-modes, the sound power can be accurately calculated in dimensionless frequency range 0< *kl* < 5. For *kl* > 5, it seems that more high order C-modes needed to estimate the accurate sound power.

**Figure 15:**
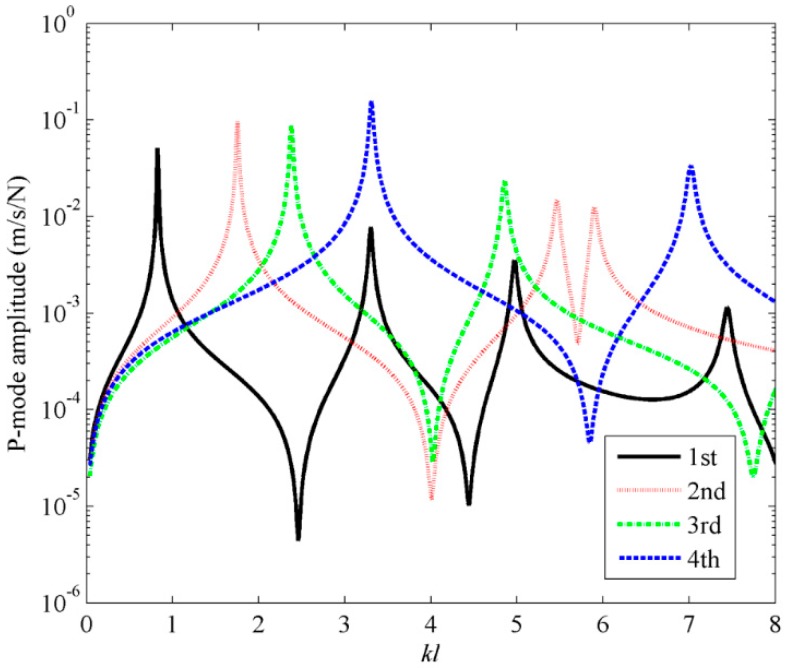
The first four C-mode amplitudes for plate under the point force excitation.

**Figure 16:**
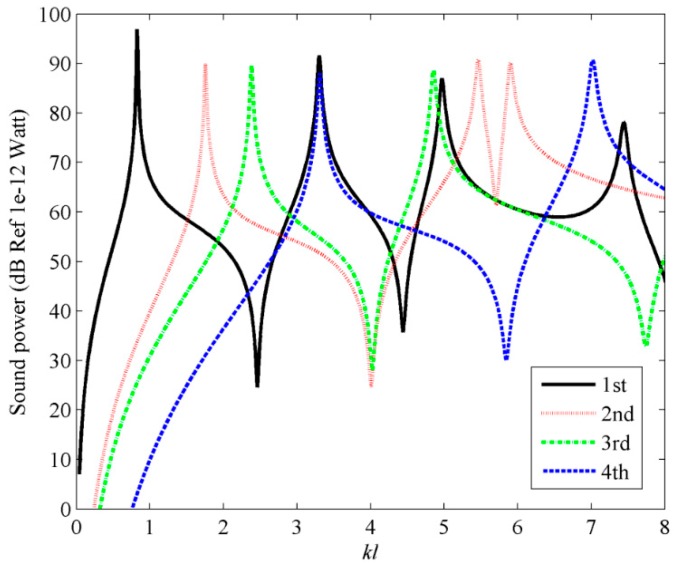
The sound power of the first four C-modes for plate under the point force excitation.

Figure 15 and Figure 16 show the first four C-mode amplitudes and the corresponding modal sound power for plate. From Figure 14, Figure 15 and Figure 16, it can be found that the sound power can be reduced significantly if the first four C-mode amplitude are controlled.

**Figure 17:**
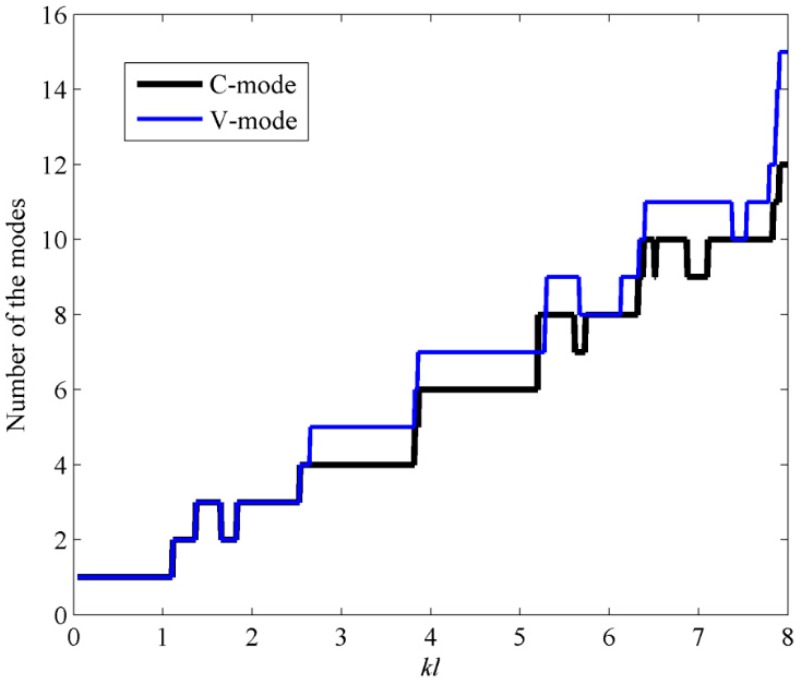
The mode number requirements by C-mode and V-mode to account for 99% of the sound power when the plate is excited by the point force.

Figure 17 shows the mode number requirements of using C-mode and V-mode, assuming the prediction precision of the sound power must exceed 99%. It can be found that the mode number requirements for C-mode and V-mode are the same for *kl* < 2.7. However, for *kl* > 2.7, compared to V-modes, less C-modes are required for the estimation of sound power.

## 5. Experimental Investigations

**Figure 18:**
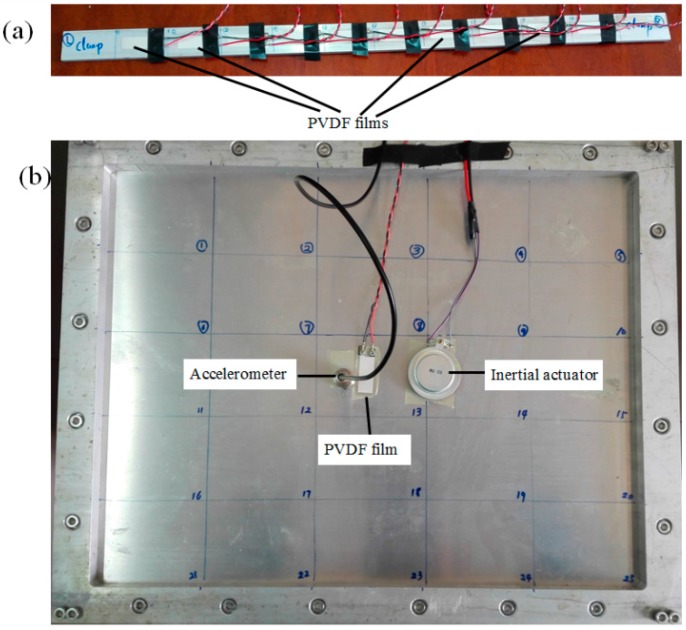
Photographs of (**a**) a beam bonded 10 PVDF films; (**b**) a clamped plate.

To further verify the proposed C-mode approach for the estimation of the sound power, a set of laboratory experiments was performed to examine its effectiveness and accuracy for real measurement data. Two applications including a clamped-clamped aluminium beam and a clamped aluminium plate are illustrated. The physical parameters of beam, plate and PVDF films are the same as numerical calculation. The experimental arrangement is shown in Figure 18. The proposed method is conducted in an ordinary room because only surface vibration information is required as the input data.

### 5.1. Sound Power Estimated from the Beam

An inertial actuator attached on the beam (at an arbitrarily chosen location *x_a_* = 150 mm), was used to provide the primary driving force for the beam. First, an accelerometer from Sinocera Piezotronics Inc. (Yangzhou, China) with the weight of 28 g, sensitivity of 50 pC/g, frequency range of 0.5–6000 Hz was used to measure the surface accelerations at 10 points, equally spaced (every 50 mm) along the length of the beam. A TST5912 dynamic signal analyzer (from Test Electron Inc., Taizhou, China) is used to acquire the frequency response functions (FRFs) between the acceleration signal of the accelerometer and the applied voltage to the inertial actuator. Then the velocity distribution under unit input voltage is obtained by the time integral of the measured FRFs. Substituting these measured velocity distribution into Equation (8), the sound power estimated by accelerometer combined V-mode theory can be obtained, which is used as a reference and will be compared to those obtained from C-modes and PVDF films. 

**Figure 19:**
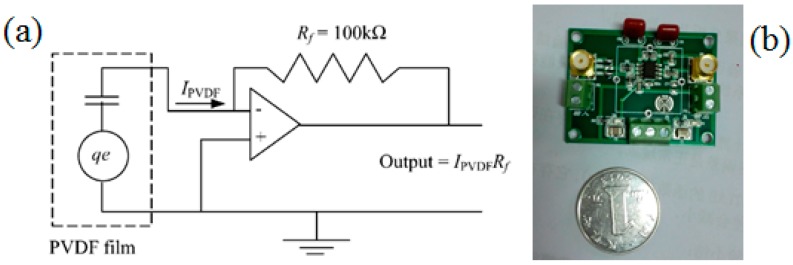
(**a**) Circuit schematic of a signal conditioner; (**b**) Photograph of the custom-built signal conditioner.

Next, the beam was equally covered with *N* = 10 segments of PVDF film (type: LDT1-28K, from Measurement Specialties Inc., Chatsworth, CA, USA), as shown in Figure 18a. Notice that the current of PVDF film needs to be measured. The PVDF film output signal was first conditioned and then measured with dynamic signal analyzer. The circuit representation of the PVDF film and the custom-built signal conditioner is shown in Figure 19. The output signal is the product of the PVDF current and the feedback resistance *R_f_*. The same measurement process as accelerometer was taken to help minimize variance errors.

**Figure 20:**
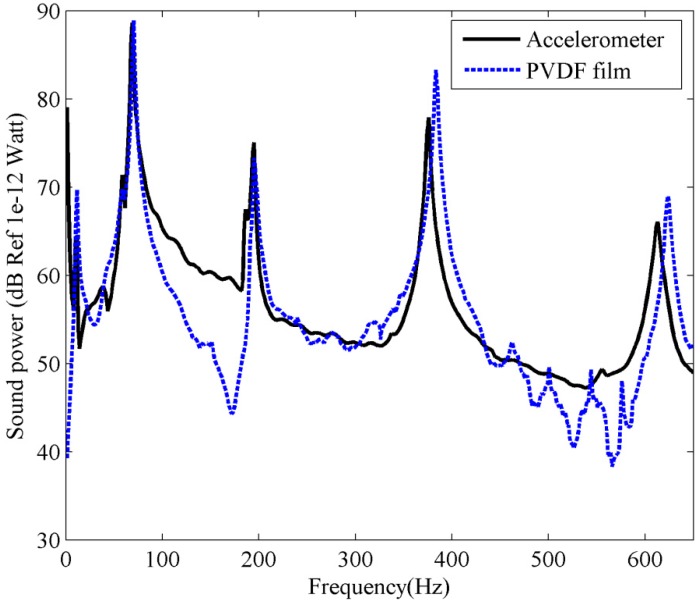
The experimental sound power for the clamped-clamped beam.

Substituting the measured PVDF current output into Equation (17), and the first three frequency independent C-modes at frequency 100 Hz (*kl* = 0.924) are used to estimate the sound power, the results are shown in Figure 20.

Notice that the reference signal used for the FRFs estimation is the voltage input to the actuator amplifier, rather than the force input to the beam, as both actuator and amplifier are part of the plant in the FRF measurements, so the estimated sound power in Figure 20 is quite different from the calculation result in Figure 7. However, good agreement can be found between accelerometer and PVDF measured sound power. It should be noted that the resonant frequencies measured by PVDF film are a little larger than those measured by accelerometer. This is because the resonant frequencies of the beam are reduced due to the weight of the accelerometer. However, when the sound power was measured using PVDF films (the accelerometer was removed from the beam), a negligible mass (in comparison to the accelerometer) is added to the beam structure, so the resonant frequencies measured by PVDF films should be more accurate than those obtained by accelerometer. This means that the proposed method using PVDF films provides a better estimation of sound power than using accelerometers.

### 5.2. Sound Power Estimated from the Plate

**Figure 21:**
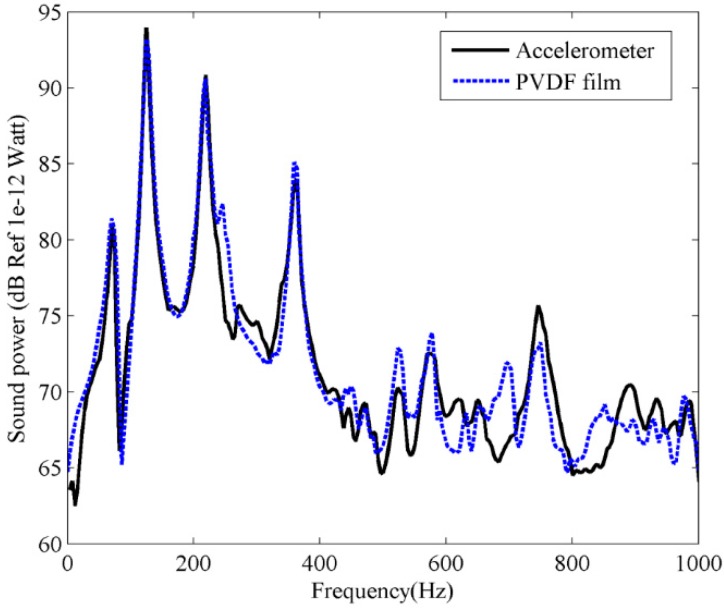
The experimental sound power for the clamped plate.

Similar to the experiment for beam, an inertial actuator attached on the plate (at an arbitrarily chosen location *x_a_* = 270 mm, *y_a_* = 170 mm), was used to provide the primary driving force. In this test, we measure the acceleration and PVDF output simultaneously, as shown in Figure 18b. This provides the PVDF and accelerometer data under the same measurement conditions. The surface vibration information at 5 × 5 locations, equally spaced along the length and width of the plate, was measured by the accelerometer and PVDF film, respectively. Using the same process as for sound power estimation for the beam, the sound power of the plate measured by accelerometer can be obtained by using Equation (8). Substituting the measured PVDF current output into Equation (17), and the first four frequency independent C-modes at frequency 200 Hz (*kl* = 1.48) are used to estimate the sound power for the PVDF film. The measured results are shown in Figure 21 and excellent agreement can be found between the accelerometer and PVDF film. It is again shown that the PVDF film with C-mode approach is able to estimate the sound power correctly in actual tests.

It should be noticed that the use of multiple PVDF films (as shown in Figure 18a for a beam) is always more precise in principle. However, from this experiment (as shown in Figure 18b), very good experimental results were achieved, despite the use of only a single removable and reusable PVDF film. This phenomenon should be further investigated. The removability and reusability have been demonstrated to PVDF film by a very simple process that used double-sided adhesive tapes. In this test, because the test plate surface kept clean and dry to maintain bond integrity, after 25 repeated peeling-bonding-measuring processes, the PVDF film showed no degradation. 

## 6. Conclusions

In this study, a new method to estimate the sound power from vibration structures by using PVDF film is proposed. The proposed method is a hybrid of vibration measurement and numerical calculation of C-modes. Differing from the classical V-modes which depend on velocity distribution of the structure, the proposed C-modes describe the sound power radiation by using the output signals of PVDF films of the vibrating structure. The PVDF films as sensors have advantages such as light weight, flexibility, and low cost. Since the proposed method does not require the measurement of sound fields, special measuring environments are not required. Numerical simulation and experimental investigation were carried out. The accuracy of the proposed method is compared with the classical V-mode approach which is widely accepted for sound power calculation and estimation [8,9].

## Figures and Tables

**Table 1:** The physical properties of PVDF film.

Parameter	PVDF Film
Length *P_x_* (mm)	30
Width *P_y_* (mm)	12
Thickness(mm)	28 × 10^−3^
Desity (Kg/m^3^)	1.78 × 10^3^
Poisson’s ratio	0.28
Young’s modulus (N/m^2^)	2–4 × 10^9^
Piezo Strain Constant *d*_31_ (m/V)	23 × 10^−12^
Maximum Operating Voltage (V/µm)	750
Relative permittivity ε/ε_0_	12
